# Computational Predictions for OCD Pathophysiology and Treatment: A Review

**DOI:** 10.3389/fpsyt.2021.687062

**Published:** 2021-10-01

**Authors:** Krisztina Szalisznyó, David N. Silverstein

**Affiliations:** ^1^Department of Neuroscience and Psychiatry, Uppsala University Hospital, Uppsala, Sweden; ^2^Theoretical Neuroscience Group, Wigner Research Centre for Physics, Hungarian Academy of Sciences, Budapest, Hungary; ^3^Agora for Biosystems, Sigtuna Foundation, Sigtuna, Sweden

**Keywords:** OCD, computational modeling, trans-diagnostic perspective, computational psychiatry, personalized treatment

## Abstract

Obsessive compulsive disorder (OCD) can manifest as a debilitating disease with high degrees of co-morbidity as well as clinical and etiological heterogenity. However, the underlying pathophysiology is not clearly understood. Computational psychiatry is an emerging field in which behavior and its neural correlates are quantitatively analyzed and computational models are developed to improve understanding of disorders by comparing model predictions to observations. The aim is to more precisely understand psychiatric illnesses. Such computational and theoretical approaches may also enable more personalized treatments. Yet, these methodological approaches are not self-evident for clinicians with a traditional medical background. In this mini-review, we summarize a selection of computational OCD models and computational analysis frameworks, while also considering the model predictions from a perspective of possible personalized treatment. The reviewed computational approaches used dynamical systems frameworks or machine learning methods for modeling, analyzing and classifying patient data. Bayesian interpretations of probability for model selection were also included. The computational dissection of the underlying pathology is expected to narrow the explanatory gap between the phenomenological nosology and the neuropathophysiological background of this heterogeneous disorder. It may also contribute to develop biologically grounded and more informed dimensional taxonomies of psychopathology.

## 1. Introduction

**Figure 1:**
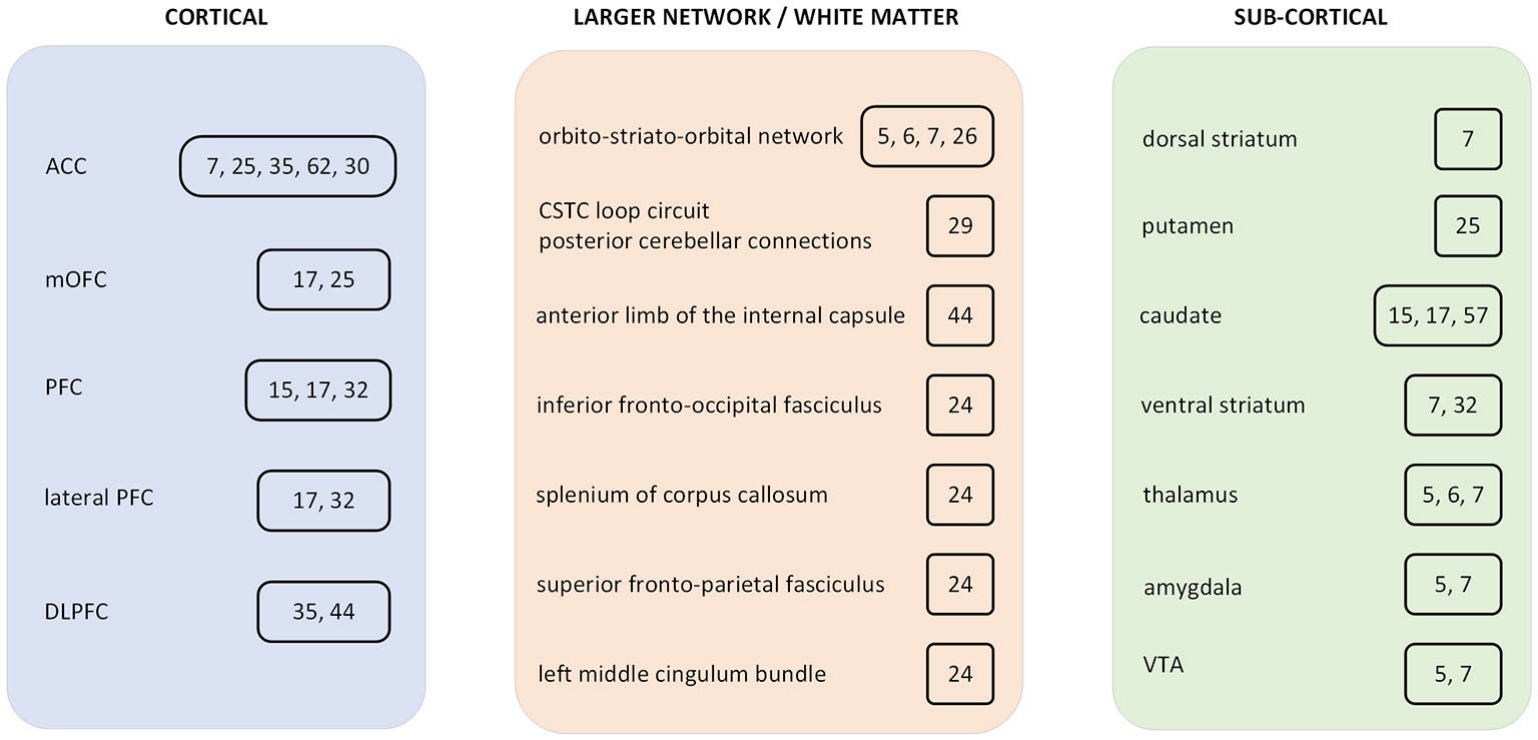
Summary diagram of brain regions which were included in obsessive compulsive disorder (OCD) computational studies. The included studies are cited in a box next to the brain region. ACC, anterior cingulate cortex; PFC, prefrontal cortex; mOFC, medial orbitofrontal cortex; DLPFC, dorsolateral prefrontal cortex; CSTC, cortico-striatal-thalamic-cortical; VTA, ventral tegmental area.

In this review, we assessed the evolution of the computational modeling efforts that aim to study some aspects of obsessive compulsive disorder (OCD) pathophysiology. The computational and theoretical investigations support the move from the currently used nosological classification toward trans-dimensional approaches (1). This trend is motivated by a necessity to gain a deeper and more biologically grounded understanding of the disease in order to develop personalized interventions. A more precisely defined micro-behavioral analysis is often able to leverage specific and more objective biomarkers than the currently used phenomenological observations in diagnostic procedures. We reviewed computational models which utilize non-linear differential equation systems, where some aspects of the pathological neural network dynamics can be represented by perturbation of the dynamical systems (2–7). Some supervised and unsupervised machine learning (ML) methods were integrated in the review, which are utilized for classification (8–13). A plethora of reinforcement learning (RL) articles and several diverse computational analysis studies are also reviewed (14–18). Both model-based and model-free RL are utilized to examine pathological aspects of goal-directed and habitual systems in OCD. Under certain circumstances, one approach may have more explanatory power than the other. However, the gap of this dichotomic separation between model-free and model-based learning approaches is perhaps narrower than suggested in earlier studies. Some recent investigations point toward more integrated forms of RL, which can exploit richer representations and can be utilized to better explain certain aspects of OCD pathology. In this review, we selected and integrated articles that utilized data-driven approaches, for example, to predict clinical outcomes or responses to treatment, as well as theory-driven attempts where the altered information processing is modeled as the cause of psychiatric symptoms at the behavioral and neuronal level (19). Table 1 summarizes the reviewed modeling/computational articles. Figure 1 is a schematic representation of some of the brain regions, which were included in the current review.

**Table 1:** Computational modeling studies on obsessive compulsive disorder (OCD) patient groups.

**Computational predictions for OCD**	**Modeling methods**	**Reference**
Over-stability from glutamatergic over-activity depth of basins of attraction↑	Coupled differential equations attractor networks	(2, 3)
Connection abnormality subtypes, periodic orbits	Coupled differential equations	(5–7)
Sequence stability ↑ network inhibition ↓	Coupled differential equations complex attractor sequences	(4)
Intermittent dynamical instability, heteroclinic cycles	Coupled differential equations	(20, 21)
Optimal STN-DBS in treatment-refractory OCD	Stochastic differential equations	(22)
Identified 4 predictors for suicide attempt	Elastic net on clinical and socio-demographic variables	(10)
Identified 24 most predictive items for remission	Random Forest on clinical data, interviews, questionnaires	(9)
Pediatric OCD treatment (ICBT) outcome	LR, Elastic net, Random Forest, SVM, linear model	(23)
Identified brain regions and discriminating sMRI patterns	Bagged SVMs for multivariate feature selection	(11)
Patients with/without sensory phenomena	LR, KNN, Random Forest, SVM on clinical data	(8)
White matter abnormalities	Multivariate SVM on DTI data	(24)
Identified 9 predictive variables for severity	SVM, naïve Bayes on genetic, neurophysiological data	(12)
Severity from mOFC, left putamen gray matter volumes	SVR on sMRI volumes	(25)
Identified 4 trans-diagnostic data-driven groups	SNF, Random Forest on behavioral, neuroimaging data	(13)
Pathological activation in orbito-striato-thalamo-orbital network	ANN with backpropagation	(26)
↑θ power in qEEG → effect of right frontal rTMS↑	ANN classifier with PSO for EEG analysis	(27)
Identified 4 compulsive/impulsive subgroups indicating severity	PCA, K-means clustering on self-report questionnaires	(28)
CSTC connections ↑ posterior cerebellar connections ↓	Riemann Kernel PCA on rsfMRI FC matrix, XGBoost	(29)
Exaggerated cingulate error signals, learning rates ↓	Q-learning fitted to fMRI prediction error responses	(30)
Sensitivity to outcome devaluation ↑	LR, RL hybrid of model-free ← → model-based	(15)
Goal-directed deficits associated with compulsivity, intrusive thought	Using online test, questionnaire data, factor analysis, LR Elastic net, RL hybrid of model-free ← → model-based	(16)
Model-free habit formation ↑ model-based control ↓ mOFC, caudate gray matter volumes ↓	Model-free SARSA(λ) TD algorithm (habit) model-based RL algorithm (goal-directed)	(17)
With higher presynaptic dopamine in ventral striatum: → model-based coding in lateral PFC ↑ → model-free coding in ventral striatum ↓	RL hybrid of model-free ← → model-based habitual ← → goal-directed	(32)
Stimulus-bound preservation ↓ punishment-driven learning ↑D_2/3_ agonists & antagonists → punishment-driven learning ↑	7 RL models using probabilistic reversal learning data Hierarchical Bayesian model selection	(33)
Treatment strategy when risk of adverse drug effects	Meta-analysis, Bayesian hierarchical model	(34)
Error control ↑ fronto-cingulate cortex ↑dACC → left-DLPFC effective connectivity ↑	DCM, Bayesian model selection on fMRI data from congruent/incongruent Stroop task	(35)
State transition uncertainty ↑over-exploratory, over-flexibility	Optimal Bayesian change-point modelBayesian selective attention model	(36, 37)
Information gathering ↑ decision threshold ↑ delayed urgency signal	Set of Bayesian generative models on sequential information gathering task (juvenile)	(38)
Dissociation between confidence and action, abandonment of historical information, reliance on prediction errors ↑	Quasi-optimal Bayesian learning model on modified predictive-inference task	(39)
4 symptom dimensions in OCD:Incompleteness, taboo thoughts, responsibility, contamination	2-level confirmatory factor analysisBayesian structural equation models	(40)
Impaired transfer across repeated decision episodesDriven by implicit memory	Bayesian multilevel drift-diffusion model on dot-motion computer tasks	(41)
On verbal recognition memory, discriminability ↓ between old and new stimuli	Bayesian multilevel drift-diffusion model on verbal computer tasks and questionnaires	(42)
Decision threshold ↑ response times ↑	Hierarchical drift-diffusion model on RDMT	(43)
Modulation of right anterior middle frontal gyrus is effective Stimulation of specific fiber pathways at lower amplitude may be superior	Tractography-activation models Electric field models of DTI-guided ALIC-NA DBS	(44)
SWN properties: β band ↓θ band with poor insight ↓	SWN graph theoretical analysis of resting-state EEG	(45)
Overly steep attractor basins	Review	(46)
Excitatory/inhibitory imbalance, inhibition not decreased	Review	(47)
Heterogeneity, local stim. of networks, factors for rTMS in OCD	Review	(48)
Brain networks in flexibility deficits	Review	(49)
↑ habit formation ← → goal-directed control ↓Compulsion → Obsession (COD)	Review	(50, 51)
Intermediate systems between model-free ← → model-based	Review	(18)
Inability to switch between goal directed ← → habitual systems	Review	(52)
Habit formation and goal-directed deficits	Review	(1)
Disruptions of complex reasoning systems	Review of juvenile OCD	(53)

*References highlighted in various colors represent method classes: 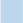 dynamical systems; 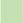 supervised and unsupervised ML algorithms; 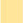 reinforcement learning approaches; 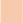 Bayesian, drift-diffusion and other methods; 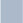 review articles, respectively. STN-DBS denotes sub-thalamic nucleus (STN) and deep brain stimulation (DBS), ALIC-NA denotes anterior limb of the internal capsule (ALIC) and the nucleus accumbens (NA). ICBT, Internet-delivered cognitive behavior therapy; LR, logistic regression; SVM, support vector machine; KNN, K-nearest neighbor; DTI, diffusion tensor imaging; mOFC, medial orbitofrontal cortex; SVR, support vector regression; sMRI, structural MRI; SNF, similarity network fusion; qEEG, quantitative EEG; ANN, artificial neural network; PSO, particle swarm optimization; PCA, principal component analysis; CSTC, cortico-striatal-thalamo-cortical; rfsMRI, resting-state fMRI; FC, functional connectivity; RL, reinforcement learning; TD, temporal difference learning; dACC, dorsal anterior cingulate cortex; DCM, dynamic causal modeling; RDMT, random-dot motion task; SWN, small world network*.

## 2. Dynamical Systems Approach

Several computational approaches utilizing dynamical systems have been developed, which can provide mechanistic insights about pathological neural dynamics in OCD. In these modeling frameworks, coupled non-linear differential equation systems were manipulated and perturbed. The solutions of these non-linear dynamical systems can exhibit a steep attractor state (e.g., fixed-point attractor), which can mimic states of perseveration, obsessions, and compulsions (2, 3, 46). Rumination or recurring chains of thought and stereotypical movement patterns were also modeled with non-linear differential equations where the solution of the dynamical system results in heteroclinic chains of meta-stable clusters and possible sequential chains of attractor basins (20, 21). Maia et al. (54) gave a comprehensive review on the neuropathological correlates and etiology of childhood and adult OCD. Verduzco-Flores and colleagues described their differential equation system as a model of working memory with increased stability of states or sequences, implicated to be associated with OCD (4). As a reflection on Verduzco-Flores' work, Maia pointed out that reduced inhibition does not map well to any known disturbance in OCD. However, what perhaps matters in the model is the balance between excitation and inhibition. Thus, the same pathological dynamics should occur with increased excitation and that would be consistent with evidence of glutamatergic hyperactivity (47, 55).

Other computational studies found that changes in the excitatory and inhibitory balance pushes a cortico-striatal-thalamo-cortical (CSTC) pathway to states of generalized hyper-activity. Certain changes in global E/I and specifically in the local inhibition may trigger network oscillations and generate hyper-activity throughout the entire CSTC pathway in OCD (5, 6). This framework was further developed and analyzed by taking into account the functional and structural network changes of the CSTC circuit in the schizo-obsessive population (7). The study predicted the importance of pathological activity propagation between the ventral and dorsal striatum, and highlighted other disruptive mechanisms in the CSTC pathway which could result in pathological repetitive behavior in this heterogeneous population.

## 3. Supervised and Unsupervised ML Approaches

Several computational studies utilizing ML techniques investigated certain aspects of neuropathophysiology and symptom phenomenology by analyzing and classifying OCD patient data. We review some of them.

A study using Random Forest decision trees found that clinically useful predictions of remission may not require an extensive battery of measures. A small set of assessments may efficiently distinguish between higher and lower risk OCD patients to inform clinical decision-making (9). Relevant predictors of suicide attempts by OCD patients were examined with Elastic net regression, a linear combination of Lasso and Ridge methods. Previous suicide planning, previous suicide thoughts, lifetime depressive episodes, and intermittent explosive disorder symptoms were found to be relevant predictors (10). Applying Support Vector Regression (SVR) identified gray matter volumes in the cortical-subcortical loops to predict OCD symptom severity. The left medial orbitofrontal cortex (OFC) and the left putamen gray matter volume were identified as neurobiological markers. The same study demonstrated that the best predictors of the “sexual/religious” OCD dimensions were the left medial OFC, right lateral OFC, and left anterior cingulate cortex (ACC) (25). Four different ML algorithms performed well as compared to multivariate logistic regression, in the prediction of treatment response to Internet-delivered cognitive behavior therapy (ICBT) for pediatric OCD treatment. The methods used were a linear model with best subset predictor selection, Elastic net (Lasso only), Random Forest, and Support Vector Machine (SVM) (23). In another integrative study, SVM and naïve Bayes methods identified predictors of diagnostic outcomes in patients with early onset OCD (12).

To identify brain regions relevant for OCD diagnosis, bagged linear SVMs were applied to structural MRI (sMRI) data for discrimination across 86 OCD patients and 86 control subjects. 39 brain regions were identified showing the largest differences between OCD patients and healthy controls and 36 of those were located in the frontal, temporal, and parietal cortices or in subcortical structures (11). A multivariate SVM method was also applied to fractional anisotropy of white matter using diffusion tensor imaging (DTI) on 28 OCD patients and 28 healthy controls. Successful discrimination was based on bilateral prefrontal and temporal regions, the inferior fronto-occipital and superior fronto-parietal fasciculi, splenium of corpus callosum, and the left middle cingulum bundle (24).

OCD is a heterogeneous disorder with varied symptom presentations, each of which may relate to distinct neuropsychological features. Traditionally, this heterogeneity was approached by using a symptom-based evaluation, but an alternative can involve focusing on underlying symptom motivations (8). Note that 60–70% of OCD patients also can experience sensory phenomena, consisting of uncomfortable sensations or perceptions that may drive compulsions. Supervised ML methods (Random Forest, SVM, and K-nearest neighbor) were tested in one set to discriminate between OCD patients and healthy controls and another set to discriminate between OCD patients with sensory phenomena, without sensory phenomena and healthy controls. All three ML methods performed better than logistic or multimodal regression on the same datasets. Decision-making measurements best distinguished between groups based on sensory phenomena (8).

With unsupervised learning, a combination of Principle Component Analysis (PCA) and a K-means clustering algorithm was utilized to separate subgroups in the compulsive-impulsive dimensions. Clustering converged to yield four subgroups: low compulsivity–low impulsivity group; two groups showing roughly equal clinical severity, but with opposing dimensions (i.e., high compulsivity and low impulsivity, and vice versa); and the fourth with both high compulsivity and impulsivity and recording the highest clinical severity. The largest cluster of individuals with OCD was characterized by high impulsivity and low compulsivity (28). The identification of these subgroups might have potential implications for OCD treatment.

A recent study based on multi-level brain imaging and behavioral data from children using the Random Forest classification algorithm identified four new brain-behavior groups cutting across neurodevelopmental disorders such as autism spectrum disorder, OCD, and attention-deficit/hyperactivity disorder (13). It was demonstrated that children within these groups had more similar profiles on brain and behavioral measures than found among conventional diagnostic groupings (13).

## 4. Reinforcement Learning: Goal-Directed and Habitual Systems

Another class of models were developed to simulate goal-directed behavior, where OCD patients may have impairment. Deficits in goal-directed control implies vulnerability for developing rigid habits (16). These models are usually computationally formalized as a type of RL (56) and can be regarded formally as dynamical systems as well (46).

Model-based RL learns to represent the environment for goal-directed predictions and allows learning to guide actions most accurately, at the expense of high computational and energy costs. Model-free RL optimizes dynamics and heuristics for habit learning without external representations and it demands less computational and memory resources, but is inflexible and generalizes poorly (15).

In healthy cohorts, individual differences in model-based learning predicted sensitivity to outcome devaluation, suggesting that an associative mechanism underlies a bias toward habit formation. But no evidence was found of a causal relationship between model-free learning and devaluation sensitivity (15).

Most previous work focused on distinguishing between only two RL systems: model-based and model-free RL (14), as prototype extremes. Recent evidence shows that there are likely several parallel systems present in the brain, which are involved in OCD pathology and their dynamics is best captured by a mixture of RL algorithms (18, 53). It has been suggested that model-free learning might simply be an imperfect formalization of habit-learning (1). A review article proposed that inflexible reliance on habit in OCD may reflect a functional weakness in the mechanism for context-appropriate dynamic arbitration between model-free and model-based decision-making (52). Thus, re-consideration is needed about this model-free/model-based dichotomy. For example, it was found that model-free spatial-motor outcome-irrelevant learning generalized across distinct state features (31, 53). In a meta-study of juvenile OCD (53), subjects had difficulties in model-based complex decision-making and set shifting. However, unlike adults, there was only limited evidence for pathologies such as distorted habit formation.

Model-based (over model-free) strategies were found to be positively correlated with gray matter volume in the ventromedial prefrontal cortex (PFC) and caudate, regions that are critical for goal-directed control (15, 17). Dysfunctional caudate hyperactivity was shown in OCD patients when performing habits (15, 57). In a healthy population, ventral striatal presynaptic dopamine levels reflected a balance in behavioral and neural signatures of model-free and model-based control. Higher presynaptic dopamine levels were associated with stronger coding of model-based information in lateral PFC and diminished coding of model-free prediction errors in ventral striatum (32).

In adults, stimulant addiction and OCD were associated with a significant shift in habit formation and this abnormality can be quantified as model-free learning. Lower gray matter volumes in the caudate, medial OFC, and lateral prefrontal cortices were associated with a greater shift toward model-free habit formation (17).

## 5. Bayesian Approaches for OCD

A plethora of studies have built on the idea that the brain implements Bayesian inference. This can be formalized in a Bayesian state-space model that aims to infer the current state of the environment by combining prior knowledge and current evidence, weighting each by its relative uncertainty. With this, learning is governed by the balance between uncertainty on state transitions and observational uncertainty (36).

Some theoretical works analyzed the assumption that OCD patients have excessive uncertainty regarding state transitions. In this case, high transition uncertainty results in increased relative weighting of prediction errors. This could explain findings of increased responses to predictable stimuli. The increased weighting of prediction errors seems more likely to be the result of high transition uncertainty than underestimation of sensory noise. Increased weighting of prediction errors are related to perceiving the world as more unstable. Further, the above alterations could account for sensory over-responsiveness in OCD, as well as the experience of intrusive thoughts. Overweighting of sensory data often implies an impairment in processing, as it leads to a failure of the use of prior information and less attenuation of sensory noise. As a further consequence, this can manifest in patients' experiences that actions were not performed correctly, obsessional thoughts, compulsions, and sensory over-responsiveness (37, 39).

Severe cognitive flexibility impairments in OCD have been described in several studies (49), although other computational works and meta-analysis pointed out that inflexibility in OCD is controversial (36, 58). A decreased reliance on the past, excessive uncertainty and an assigned lower weight to prior experience has been shown to lead to over-exploratory behavior. Also, OCD patients require longer response times, higher decision boundaries and more evidence in perceptual contexts with high uncertainties (43). Somewhat counterintuitively, OCD symptoms correlated with over-flexibility in another set of computational studies (36). Excessive uncertainty and distrust of past experiences rather than perseveration were identified and these results might challenge pre-conceptions of OCD as a disorder of inflexibility (36).

In a combined experimental and computational study, it was shown that OCD patients develop an accurate internal model of the environment but they use it less to guide behavior. This suggests a cognitive architecture that separately interprets the environment independently of performance (39). Different memory systems separately influence repeated decisions. In a study of perceptual dot motion decisions, Solway et al. found that both the actual choice made during the first decision episode as implicit memory and the choice people explicitly remember making influenced the subsequent decision. Transfers specifically driven by implicit memory were reduced in individuals with higher levels of OCD symptoms (41). Verbal recognition memory was also investigated as a function of OCD symptoms, using a drift-diffusion model selected with model evidence using a multi-level Bayesian framework (42). It was found that discriminability defined as how well one is able to tell the old vs. new stimuli apart was reduced as a function of OCD symptoms, and that the degree of impairment was larger for easier recognition decisions (42).

## 6. Additional Computational Analysis of Neuropathological Correlates

Enhanced activation in the fronto-cingulate system in OCD patients and task-related modulation of effective connectivity from the dorsal anterior cingulate cortex (ACC) to left dorsolateral PFC was demonstrated by using dynamical causal modeling (DCM) on patient fMRI data. These findings implicated an overactive error control system in OCD (35). Another method was utilized to characterize patients with OCD based on resting-state fMRI. The Riemann kernel PCA method extracted features from functional connectivity matrices and demonstrated stronger connections between basal ganglia and cortex and weaker cerebellum-related connections in OCD (29).

### 6.1. Insight

Patient insight in OCD is crucial. The diagnostic status of poor insight is ambiguous but is a key clinical factor that influences therapy outcome (59). Poor insight has been associated with earlier age-at-onset, longer duration of illness, and a more chronic course of OCD (60). Checking-related uncertainty was correlated with the level of insight in OCD patients (61). Information gathering was found to be related to indecisiveness, but not symptom severity in OCD (38). This absence of a correlation with symptom severity was implicated to be caused by an imprecise estimate of the OCD severity, which was related to a lack of insight in juvenile OCD (38).

OCD patients with good and poor insight (OCD-GI and OCD-PI) have partly distinct brain structural alterations (62). OCD-PI patients have decreased cortical thickness in the left superior frontal gyrus, left anterior ACC, and right inferior parietal gyrus, compared to both OCD-GI and healthy controls (62). It was also indicated that the OCD-GI group had significantly increased functional connectivity between the right anterior insula (AI) ← → left dorsal anterior cingular cortex (dACC) than healthy controls (63). The connectivity alterations between the AI ← → OFC and AI ← → ACC may be important neural correlates of insight in OCD and even in schizophrenia (7). Alterations have been demonstrated at the theta (θ) EEG band in a small-world network framework and these changes existed only in the OCD-PI patients but not in the OCD-GI patients. Thus, poor insight OCD may be associated with disruptive functional integrity in the brain functional network in the theta band (45).

### 6.2. Co-morbidity and Trans-dimensional Analysis

Trans-dimensional biologically grounded approaches to OCD symptoms are supported by the obvious existence of OCD sub-types. A shift from a categorized disease framework to a dimensional one may enable more personalized treatment choices (16). We list some examples of such approaches.

RL models were able to capture certain behavioral microstructure differences between stimulant use disorder (SUD) and OCD. Stimulus-bound perseveration is a measure of how a subject is responding to a repeated stimulus, irrespective of outcome. This measure was found to be significantly increased in SUD, but decreased in OCD, compared to controls. Individuals with SUD exhibited reduced reward-driven learning, while both the SUD and OCD groups showed increased learning from punishment. Dopamine receptor *D*_2/3_ agonists and antagonists had similar effects on OCD groups, as both increased punishment-driven learning (33). In addition, a pharmacological fMRI study of RL has shown an abnormally increased signaling of prediction errors in the anterior ACC. This effect was reduced by both a *D*_2/3_ agonist and an antagonist (30). Modeling results did not demonstrate the same effects but did show a marginally significant reduction in prediction error learning rates in OCD patients (30).

Another aspect of behavioral microstructure was analyzed by 2-level factor modeling in OCD patients. This modeling study found that heterogeneous symptoms (as quantified, e.g., in Yale-Brown Obsessive Compulsive Scale) reflect four underlying symptom dimensions with deviations from previous results (40).

Obsessions and compulsions might independently contribute to the pathophysiology (1, 16). An alternative possibility posits that rather than goal-directed avoidance behaviors, compulsions derive from manifestations of excessive habit formation (50, 51), thus obsessive thoughts may develop as a result of compulsive behavior. It has even been suggested that the acronym OCD be rearranged to COD (50). Compulsivity and impulsivity might be only partially independent dimensions, considering that patients with substance abuse can transition from impulsivity to compulsivity (16). A “Compulsive Behavior and Intrusive Thought” dimension has been described as deficits in goal-directed control and presented in multiple psychiatric disorders such as OCD, addiction, and eating disorders (16).

The neuropathophysiology of co-morbid OCD and schizophrenia was examined in a phenomenological computational model (7). It was found that cortical self-inhibition alterations (e.g., SSRI treatment) together with dopaminergic input to the striatum (e.g., anti-dopaminergic medication) has non-trivial complex effects on the network oscillatory behavior, with an optimal modulatory window. Also, the modeling results predicted that as a consequence of over-compensation of the primary pathology, emergence of the other disorder might occur (7).

### 6.3. Personalized Computational Approaches

The clinical implications of certain computational results suggest possible development of personalized medicine to identify and optimize specific therapies for individual OCD patients. We list some of those efforts. Pre-treatment functional connectivity patterns within the default mode network and visual network significantly predicted the effect of cognitive behavioral therapy (CBT) and post-treatment OCD severity. These networks were stronger predictors than pre-treatment clinical scores (64). Abnormally strong cingulate signaling was measured using fMRI during reward processing with OCD patients. Bidirectional re-mediation by dopaminergic modulation suggests that exaggerated cingulate error signals in OCD may be of dopaminergic origin (30).

Transcranial magnetic stimulation (TMS) has shown promise as an adjunct treatment for the symptoms of OCD (48). Quantitative EEG was found to be helpful for predicting TMS treatment response for OCD patients. Using artificial neural network (ANN) classifiers with Particle Swarm Optimization (PSO) it was found that repetitive TMS responders had higher pre-treatment theta band power at all electrodes than did the non-responders (27).

Therapy refractory OCD patients have benefited from deep brain stimulation (DBS). Optimal therapeutic results are associated with the activation of distinct fiber pathways. The stimulation of the right anterior middle frontal gyrus (DLPFC) has shown a positive response. Focused stimulation of specific fiber pathways, which allows stimulation with lower amplitudes, may be superior to activation of a wide array of pathways, typically associated with higher stimulation amplitudes (44). Closed-loop neuromodulation is an emerging field in DBS. Model-based prediction was proposed for an optimal sub-thalamic nucleus (STN) DBS on treatment-refractory OCD with a combination of a stochastic dynamical model and microelectrode recording datasets (22).

A recent meta-analysis using a Bayesian hierarchical model framework examined adverse effects of selective serotonin reuptake inhibitors (SSRIs) and serotonin-norepinephrine reuptake inhibitors (SNRIs) treatments in pediatric OCD and anxiety disorders. It was found that compared with SNRIs, SSRIs are more likely to produce activation such as insomnia, irritability, hyperactivity, and impulsivity. The results suggested that although SSRIs are superior to SNRIs and the treatment of choice, for those patients who become activated on SSRIs, SNRIs might represent a good second choice given their reported efficacy and lower risk of activation (34).

## 7. Limitations

There are computational contributions that were not included in the current work. The contents of the obsessive-compulsive symptoms (sexual, religious, aggressive, contamination) were only partially explored. Computational work with brain histology was also excluded. We did not include comprehensive aspects of the developmental trajectories of the disease (age of onset, etc.). Further reviews are necessary to follow and categorize this rapidly growing field.

## 8. Conclusion

To summarize, we reviewed some of the computational modeling efforts which were developed to explain certain aspects of OCD pathophysiology and symptomology. These models span from mechanistic dynamical systems approaches, across ML techniques which aim to integrate and classify patient data (including supervised, unsupervised models, RL), to include Bayesian model selection frameworks. We related the modeling evidence and results to diagnostic procedures, co-morbid states, and therapeutical consequences. In conclusion, computational psychiatry has powerful methods, which can arm psychiatrists with more quantitative tools (46). Although it is challenging to move from a phenomenologically based thought process to a dynamical approach, we claim that a phase transition in understanding psychiatric disease as dynamical pathologies is inevitable. To this end, computational/theoretical frameworks have been synthesized to capture how OCD symptoms can be further analyzed from a trans-diagnostic and computational perspective (1).

## Author Contributions

All authors listed have made a substantial, direct and intellectual contribution to the work, and approved it for publication.

## Funding

KS was supported by ALF, RUFU, Märta and Nasvells Stiftelse research grants provided by the Uppsala University Hospital. Support for DS was provided by a joint grant from the John Templeton Foundation and the Fetzer Institute.

## Author Disclaimer

The opinions expressed in this publication are those of the author(s) and do not necessarily reflect the views of the John Templeton Foundation or the Fetzer Institute.

## Conflict of Interest

The authors declare that the research was conducted in the absence of any commercial or financial relationships that could be construed as a potential conflict of interest.

## Publisher's Note

All claims expressed in this article are solely those of the authors and do not necessarily represent those of their affiliated organizations, or those of the publisher, the editors and the reviewers. Any product that may be evaluated in this article, or claim that may be made by its manufacturer, is not guaranteed or endorsed by the publisher.
